# Age Estimation from Lateral Cephalograms Using Deep Learning: A Pilot Study from Early Childhood to Older Adults

**DOI:** 10.3390/jcm14197084

**Published:** 2025-10-07

**Authors:** Ryohei Tokinaga, Yuichi Mine, Yuki Yoshimi, Shota Okazaki, Shota Ito, Saori Takeda, Saki Ogawa, Tzu-Yu Peng, Naoya Kakimoto, Kotaro Tanimoto, Takeshi Murayama

**Affiliations:** 1 Department of Medical Systems Engineering, Graduate School of Biomedical and Health Sciences, Hiroshima University, Hiroshima 734-8553, Japan; m251922@hiroshima-u.ac.jp (R.T.); s.okazaki@scu.ac.jp (S.O.); d252710@hiroshima-u.ac.jp (S.T.); murayatk@hiroshima-u.ac.jp (T.M.); 2 Project Research Center for Integrating Digital Dentistry, Hiroshima University, Hiroshima 734-8553, Japan; 3 Department of Orthodontics and Craniofacial Developmental Biology, Graduate School of Biomedical and Health Sciences, Hiroshima University, Hiroshima 734-8553, Japan; yukimihsoy@hiroshima-u.ac.jp (Y.Y.); shota0313@hiroshima-u.ac.jp (S.I.); osakitty@hiroshima-u.ac.jp (S.O.); tkotaro@hiroshima-u.ac.jp (K.T.); 4 AIT Center, Sapporo City University, Sapporo 060-0061, Japan; 5 School of Dentistry, College of Oral Medicine, Taipei Medical University, Taipei 11031, Taiwan; 6 Department of Oral and Maxillofacial Radiology, Graduate School of Biomedical and Health Sciences, Hiroshima University, Hiroshima 734-8553, Japan; kakimoto-n@hiroshima-u.ac.jp

**Keywords:** artificial intelligence, deep learning, lateral cephalograms, age estimation

## Abstract

**Background/Objectives**: The purpose of this study is twofold: first, to construct and evaluate a deep-learning model for automated age estimation from lateral cephalograms spanning early childhood to older adulthood; and second, to determine whether sex-specific training improves predictive accuracy. **Methods**: This retrospective study examined 600 lateral cephalograms (ages 4–63 years; 300 female, 300 male). The images were randomly divided into five cross-validation folds, stratified by sex and age. An ImageNet-pretrained DenseNet-121 was employed for age regression. Three networks were trained: mixed-sex, female-only, and male-only. Performance was evaluated using mean absolute error (MAE) and the coefficient of determination (*R*^2^). Grad-CAM heatmaps quantified the contributions of six craniofacial regions. Duplicate patients were excluded to minimize sampling bias. **Results**: The mixed-sex model achieved an MAE of 2.50 ± 0.27 years, an *R*^2^ of 0.84 ± 0.04, the female-only model achieved an MAE of 3.04 ± 0.37 years and an *R*^2^ of 0.82 ± 0.04, and the male-only model achieved an MAE of 2.29 ± 0.27 years and an *R*^2^ of 0.83 ± 0.04. Grad-CAM revealed dominant activations over the frontal bone in the mixed-sex model; the occipital bone and cervical soft tissue in the female model; and the parietal bone in the male model. **Conclusions**: A DenseNet-121-based analysis of lateral cephalograms can provide a clinically relevant age estimation with an error margin of approximately ±2.5 years. Using male-only model slightly improves performance metrics, and careful attention to training data distribution is crucial for broad applicability. Our findings suggest a potential contribution to forensic age estimation, growth and development research, and support for unidentified deceased individuals when dental records are unavailable.

## 1. Introduction

Age estimation of individuals based on medical and dental images is an important issue in forensic medicine, such as victim identification after major disasters and criminal investigations [1,2,3]. Traditional methods of age estimation from radiographs have relied on expert assessment of morphological features (e.g., stage of tooth development and cervical spine maturity in lateral cephalograms), but such methods are time-consuming and subjective [4,5,6]. Recent advances in deep learning have enabled automated, objective approaches that learn age-related patterns from large image datasets and show great promise [7,8,9].

Panoramic radiographs have been widely investigated for age estimation due to their broad view of all teeth and jaws. Deep learning models on panoramic radiographs have achieved high accuracy over wide age ranges [9]. For example, in a recent study, the DentAge model [10] was trained on 21,007 panoramic images (ages 4–97) and reported a mean absolute error (MAE) of 3.12 years on the test set. Notably, the model performed best in younger adults (e.g., MAE ~1.94 years for ages 10–20), while errors increased in the very elderly (MAE ~13.4 years for ages 90–100). These results demonstrate that, despite the geometric distortions inherent in panoramic radiographs, deep networks can learn reliable age predictors from dental structures. However, panoramic images primarily capture dental and jaw features and lack other anatomical cues of aging.

Lateral cephalograms capture the craniofacial complex in a single, standardized projection that depends on the equipment and imaging conditions [11,12,13]. When the head is properly positioned in the cephalostat and the structures of interest lie close to the midsagittal plane, linear distances can be corrected with a single scale factor, and angular relationships are essentially considered to reflect real objects [14,15]. In contrast, panoramic radiography introduces anisotropic distortion. This distortion limits the validity of absolute measurements [16,17]. In addition to geometric fidelity, lateral cephalograms depict the soft tissue profile, skull base, and cervical spine in addition to the dentition. Deep learning work by Zhang et al. [18] showed that age-related salience on lateral cephalograms is distributed across the teeth, maxillofacial skeleton, and cervical vertebrae. This distribution supports the multiple information available in cephalograms. These skeletal structures continue to undergo degenerative changes throughout adulthood [19,20,21]. Such changes provide non-dental clues to aging that are absent or poorly visualized on panoramic radiographs.

Therefore, the purpose of this study was to construct and evaluate a deep learning model for automated age estimation from lateral cephalograms across a wide age range from early childhood to older adults. Specifically, we aimed to: (1) assess the accuracy of age prediction using a DenseNet-121 architecture on lateral cephalograms, (2) compare the performance between sex-specific models and a mixed-sex model, and (3) visualize the anatomical regions contributing to age estimation using a saliency map to guide future forensic and clinical applications.

## 2. Materials and Methods

### 2.1. Study Overview and Ethical Approval

A retrospective observational study was conducted using lateral cephalometric radiographs acquired for routine orthodontic diagnoses between January 2019 and December 2023. The protocol was approved by the Ethical Committee for Epidemiology of Hiroshima University (Approval Number: E2022-0211), and the requirement for written informed consent was waived owing to the retrospective nature of the investigation. The study complied with the principles outlined in the Declaration of Helsinki.

### 2.2. Dataset and Experimental Design

The dataset included 600 lateral cephalograms (300 male and 300 female) obtained using a cephalometric scanner (CX-150W; Asahi Roentgen Ind. Co., Ltd., Kyoto, Japan). All lateral cephalograms were acquired under standardized positioning using a cephalostat. By design, lateral cephalometric radiography collapses 3D anatomy into a 2D projection, resulting in right–left superimposition of craniofacial structures in every image. Images obtained from the same patient were not included. The image resolution was 1648 × 1980 pixels. Images with a lot of empty space have been cropped to leave the identifiable parts of the subject. The ages of the subjects ranged from 4 to 63 years old (mean ± standard deviations = 17.1 ± 10.5 years). The age distribution of the subjects is provided in Table 1.

Three experimental conditions were evaluated: a combined set of male and female data (the mixed-sex model), a set of male-only data, and a set of female-only data. Model validation was performed using five-fold cross-validation [22] for all three conditions. For each condition, the respective dataset was randomly partitioned into five equal folds. Each fold served as the test set once, and the remaining four folds were used for training. For the female-only and male-only conditions, the 300 images were divided into a training set of 240 images and a test set of 60 images. For the mixed-sex condition, the dataset was divided into a training set of 480 images and a test set of 120 images. This process was repeated five times for each condition to obtain comprehensive performance metrics and sex-specific analyses across all data samples. The performance metrics represent the mean ± standard deviations across the five test folds for each setting.

### 2.3. Network Architecture and Deep Learning

All procedures were conducted on a computer equipped with an Intel Core i5-10200H 2.40 GHz CPU (Intel, Santa Clara, CA, USA), 16 GB of RAM, and an NVIDIA GeForce RTX 3060 GPU (NVIDIA, Santa Clara, CA, USA), using Python software (version 3.8.15), Keras framework (version 2.4.3) and TensorFlow (version 2.4.0).

Age was regressed using the pre-trained DenseNet-121 [23] model on ImageNet. The final network architecture consisted of the pre-trained DenseNet-121 base model, followed by a global average pooling layer. A dense layer containing 1024 units with a Rectified Linear Unit activation function was added, followed by a single output neuron with linear activation for regression. The model was compiled using the RMSprop optimizer with a learning rate of 1 × 10^−5^ and momentum of 0.9. Mean squared error was used as the loss function and mean absolute error was employed as the evaluation metric during training. All input images were resized to 256 × 256 pixels using OpenCV library. Image normalization was performed by subtracting the dataset mean and dividing by the dataset standard deviation.

Training was conducted with a batch size of 10 for up to 500 epochs. Early stopping was implemented with a patience level of 10 epochs and monitoring of the validation loss. The learning rate was reduced when the validation loss plateaued, with a reduction factor of 0.3 and a patience level of 3 epochs.

### 2.4. Performance Metrics

The performance of the age-estimation deep learning model was evaluated using lateral cephalograms and two complementary statistics: MAE and the coefficient of determination (*R*^2^). Of these, *MAE* quantifies the average absolute discrepancy between the model’s age estimate and the subject’s chronological age, offering an intuitive measure of predictive accuracy. It is defined as

(1) $$MAE=\frac{1}{n}\sum_{i=1}^{n}\left|y_{i}-y_{i}^{\prime }\right|,$$

where *yi* represents the true age derived from each participant’s date of birth, and *yi*′ is the corresponding age inferred by the deep learning model from the cephalogram.

Gradient-weighted class activation mapping (Grad-CAM) [24] was implemented to visualize the regions contributing to age predictions. The technique used gradients from the final convolutional layer of the DenseNet-121 architecture. Gradients of the predicted age were calculated with respect to feature maps in the final convolutional layer. The resulting activation maps were resized to match the dimensions of the original images and superimposed onto the original images with 50% transparency using the jet colormap. Saliency maps were generated for all test images.

We used a modified version of the hit rate metric reported by Saporta et al. [25] to quantify the overlap of saliency maps across test images. This metric was used to determine whether saliency maps existed near lesions and was not intended to strictly identify the overall extent of pathology. Using this metric, we quantified hit rates for multiple regions in the saliency maps of test images: (1) frontal bone, (2) parietal bone, (3) occipital bone, (4) soft tissues of the neck, (5) cervical spine, and (6) maxilla, mandible, and teeth.

### 2.5. Statistical Analysis

Statistical analyses were performed using IBM SPSS Statistics 27 (IBM Corp., Armonk, NY, USA). The Kruskal–Wallis test was employed as a non-parametric analysis, followed by Dunn’s test for pairwise comparisons. These analyses were used to evaluate differences among three independent groups. The values are expressed as means ± standard deviations. Significance was set at *p* < 0.05.

## 3. Results

**Figure 1:**
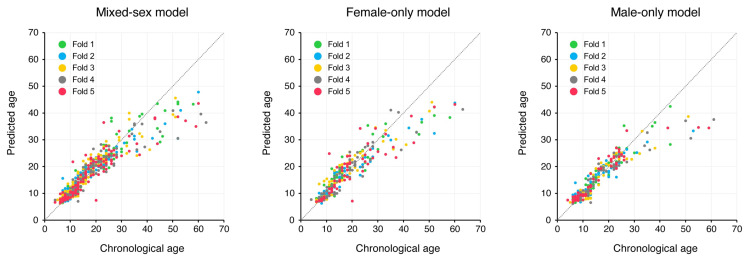
Scatter plot of the ages of images in each test dataset in five-fold cross-validation and the ages predicted by DenseNet-121.

Regression analyses were performed using DenseNet-121 models. The predicted outcomes are plotted against the chronological ages in Figure 1. Model performance across the five test folds is summarized in Table 2. The male-only model achieved the lowest MAE value (2.29 ± 0.27 years). In contrast, the mixed-sex model yielded the highest *R*^2^ (0.84 ± 0.04). The female-only model showed an intermediate *R*^2^ value (0.82 ± 0.04) and the largest MAE value (3.04 ± 0.37 years).

**Figure 2:**
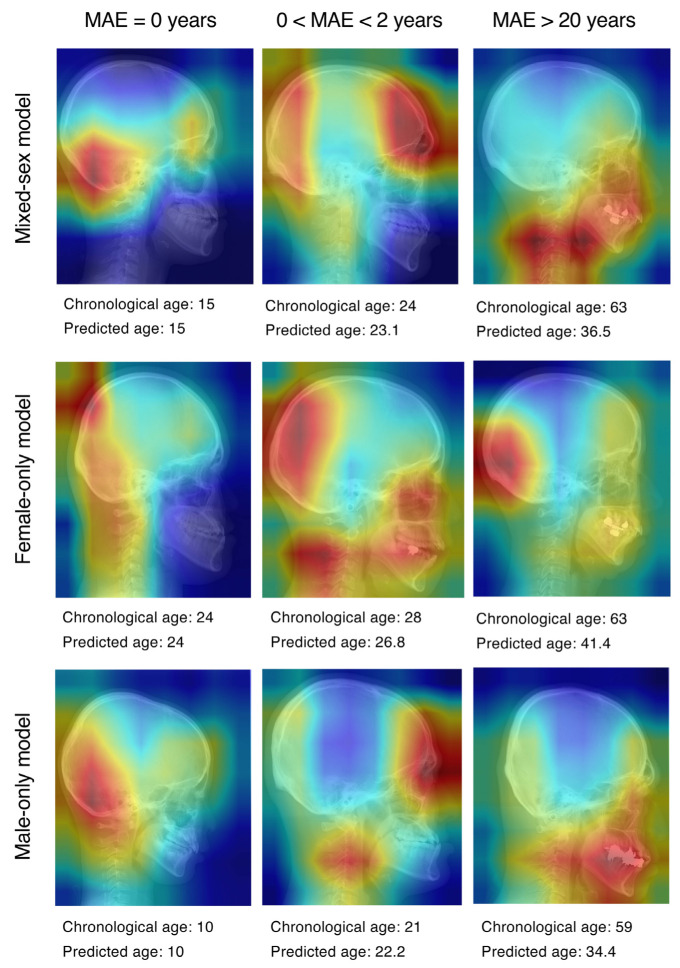
Grad-CAM saliency map visualization of DenseNet-121 age-prediction models. The columns correspond to the MAE between chronological age and predicted age: (**left**) exact prediction (MAE = 0 years); (**center**) small error (0 < MAE < 2 years); (**right**) large error (MAE > 20 years). The rows show the three DenseNet-121 models: mixed-sex (**top**), female-specific (**middle**), and male-specific (**bottom**). Warmer colors in the color map denote a higher contribution to the regression output. MAE: mean absolute error.

**Figure 3:**
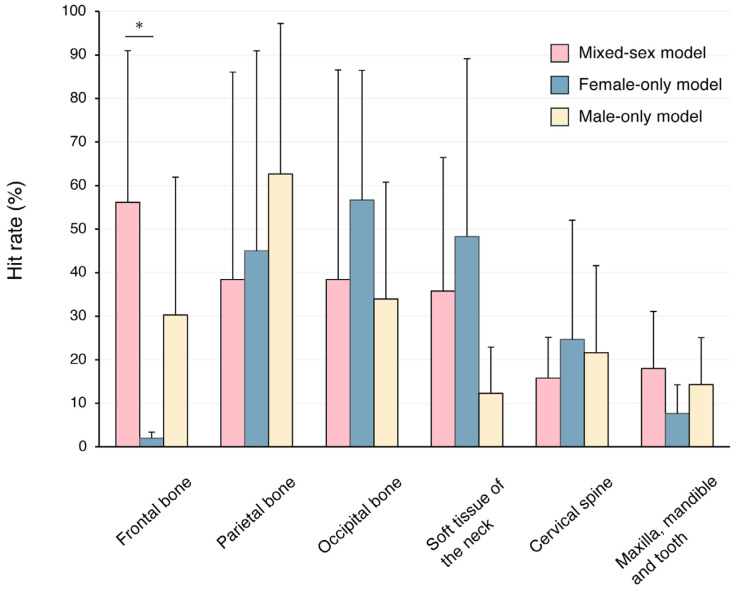
Quantitative hit-rate analysis of Grad-CAM saliency maps for six craniofacial regions (frontal bone, parietal bone, occipital bone, cervical spine, soft tissue of the neck, and maxilla/mandible/teeth) across the three DenseNet-121 models (mixed-sex, male-specific, female-specific). Data represent means ± standard deviations. Asterisks indicate statistically significant differences. * *p* < 0.05.

Figure 2 shows the results of representative Grad-CAM saliency map. A quantitative evaluation of the Grad-CAM saliency maps revealed systematic differences in the anatomical regions emphasized by each network (Figure 3). In the mixed-sex model, the frontal bone accounted for the highest proportion of peak activations, followed by the occipital bone. The female-only model focused on the occipital bone and the soft tissue of the neck. The male-only model most frequently focused on the parietal bone. The Kruskal–Wallis test and Dunn’s post hoc comparison revealed that the activation ratio in the female model was significantly lower than in the mixed-gender model in the frontal bone (*p* < 0.05). However, no significant differences were observed in other areas.

## 4. Discussion

In this pilot study, we evaluated a deep learning model, DenseNet-121, for estimating chronological age from lateral cephalograms across early childhood through older adults. We observed slight differences in performance when the model was trained and applied separately for males and females as opposed to using a mixed-sex model on the combined dataset. We also found that the uneven age distribution in our study sample (with a large number of adolescents and relatively few older individuals) affected the accuracy and generalizability of age prediction.

In our analysis, the male-only model yielded slightly higher performance metrics than the mixed-sex model in MAE. Separating the training by sex may have enabled the network to more effectively capture sex-related craniofacial growth patterns, particularly during puberty, when males and females exhibit different developmental trends. For example, adolescent males typically experience later and more pronounced mandibular growth spurts than females [26,27]. Conversely, the female-only model did not achieve the performance metrics of the mixed-sex model. When training sex-specific models, we expected the signals to remain undiluted by sex differences. However, this study could not provide clear answers to these questions. Meanwhile, it was suggested that the age distribution of the dataset affects model performance. At our hospital, most of the cephalograms were collected from patients in their 10s, with only 10% of the samples being from patients aged 30 or older. This imbalance may have caused the model to skew toward the most frequently observed age group. In fact, the scatter plot of predicted versus chronological age shows that the model accurately estimated the ages of children and adolescents, who made up the majority of the training data. However, the model tended to have increased errors in the extreme age ranges. Specifically, the model tended to underestimate the ages of older adults. This behavior is consistent with other deep learning studies that have noted performance declines in age groups with insufficient training data [10,18,28].

Our DenseNet-121 model achieved an MAE of 2.50 ± 0.27 years on 600 mixed-sex lateral cephalograms spanning 4–63 years. Despite notable differences in modality, sample size, and age coverage, its error rate is comparable when benchmarked against three representative reports. Mu and Li [29] trained four ImageNet-pretrained transfer learning models on 3000 orthopantomograms from individuals aged 12 to 71 years and reported an optimal MAE of 2.83 years using EfficientNet-B5. Fan et al. [30] introduced a semi-supervised convolutional neural network (CNN) and transformer network that was trained using 15,195 orthopantomograms from individuals aged 16 to 50. They reported an MAE of 2.61 years on external testing. Although experimental conditions differed, despite their dataset being more than 25 times larger than our study, their absolute error was nearly identical to ours. This suggests that skull projections may provide abundant age-related information even to compact CNNs. Zhang et al. [18] analyzed 14,142 cephalograms from subjects aged 4 to 40 years and demonstrated that MAEs increased from 1.3 years in children to over 3 years in adults. They also presented Grad-CAM maps that identified teeth, the maxillofacial skeleton, and the cervical vertebrae as critical regions. Our results showed that the response of the frontal bone was significantly lower in the female-only model. Compared to panoramic radiography, which uses a focal trough to emphasize the dental arches, but is susceptible to magnification, ghosting, and distortion related to positioning, lateral cephalograms profile the craniofacial skeleton at the expense of per-tooth detail due to bilateral superimposition. This trade-off likely contributed to the lower hit rate in the maxilla/mandible/teeth region of interest.

This pilot study has several limitations. First, the sample size was relatively small, particularly in subgroups such as older adults. Only a few subjects over the age of 40 were included, which limits the model’s ability to identify age-related features in older adults and reduces confidence in predictions for this demographic. Second, this study was a single-center study of a Japanese population, and cephalograms were obtained using one device. While this homogeneity helped control the experimental conditions, it may also mean that the model learned population- or device-specific patterns. The model’s performance may differ in other ethnic groups or when using cephalograms from different clinics with varying equipment and image quality. Third, in this preliminary work, we evaluated only one deep learning architecture (DenseNet-121). It is possible that other network architectures or ensembles could offer greater accuracy.

## 5. Conclusions

In summary, this study demonstrates the feasibility of using deep learning to predict individual’s chronological from lateral cephalograms. Using male-only model slightly improves performance metrics, and careful attention to training data distribution is crucial for broad applicability. Our findings contribute to the growing body of evidence showing that advanced deep learning models can automatically extract meaningful biological age information from medical images. In orthodontic practice, automated age estimation could augment growth-related assessments and scheduling and reduce reliance on subjective staging. In forensic workflows, postmortem lateral skull or cephalometric radiographs, when paired with ante mortem counterparts, may aid in human identification, alongside other evidence. Future studies will focus on improving age estimation performance based on cranial radiographs using large-scale, multicenter datasets that include more elderly subjects and evaluating different network architectures.

## Figures and Tables

**Table 1:** The distribution of the subjects by age (years).

	0–9	10–19	20–29	30–39	40–49	50–59	60–65	Total
Female	68	145	50	18	9	7	3	300
Male	84	121	74	11	3	6	1	300
Total	152	266	124	29	12	13	4	600

**Table 2:** Performance metrics (mean ± standard deviations) of the DenseNet-121.

	Mixed-Sex Model	Female-Only Model	Male-Only Model
MAE	2.50 ± 0.27	3.04 ± 0.37	2.29 ± 0.27
*R* ^2^	0.84 ± 0.04	0.82 ± 0.04	0.83 ± 0.04

MAE: mean absolute error, *R*^2^: coefficient of determination.

## Data Availability

The data presented in this study are available on request from the corresponding author. The data are not publicly available due to ethical restrictions.

## Abbreviations

The following abbreviations are used in this manuscript:

MAE	mean absolute error
Grad-CAM	gradient-weighted class activation mapping
CNN	convolutional neural network

## References

[1] Pittayapat P., Jacobs R., De Valck E., Vandermeulen D., Willems G. (2012). Forensic odontology in the disaster victim identification process. J. Forensic Odontostomatol..

[2] Ruder T.D., Kuhnen S.C., Zech W.D., Klaus J.B., Lombardo P., Ith M. (2023). Standards of practice in forensic age estimation with CT of the medial clavicular epiphysis-a systematic review. Int. J. Leg. Med..

[3] Khatam-Lashgari A., Harving M.L., Villa C., Lynnerup N., Larsen S.T. (2024). Forensic age estimation of the knee by post-mortem DR, CT, and MR imaging: A comparative study. Int. J. Leg. Med..

[4] Nestman T.S., Marshall S.D., Qian F., Holton N., Franciscus R.G., Southard T.E. (2011). Cervical vertebrae maturation method morphologic criteria: Poor reproducibility. Am. J. Orthod. Dentofacial. Orthop..

[5] De Donno A., Angrisani C., Mele F., Introna F., Santoro V. (2021). Dental age estimation: Demirjian’s versus the other methods in different populations. A literature review. Med. Sci. Law.

[6] Khanagar S.B., Albalawi F., Alshehri A., Awawdeh M., Iyer K., Alsomaie B., Aldhebaib A., Singh O.G., Alfadley A. (2024). Performance of Artificial Intelligence Models Designed for Automated Estimation of Age Using Dento-Maxillofacial Radiographs-A Systematic Review. Diagnostics.

[7] Bağ İ., Bilgir E., Bayrakdar İ.Ş., Baydar O., Atak F.M., Çelik Ö., Orhan K. (2023). An artificial intelligence study: Automatic description of anatomic landmarks on panoramic radiographs in the pediatric population. BMC Oral. Health.

[8] Wesp P., Schachtner B.M., Jeblick K., Topalis J., Weber M., Fischer F., Penning R., Ricke J., Ingrisch M., Sabel B.O. (2024). Radiological age assessment based on clavicle ossification in CT: Enhanced accuracy through deep learning. Int. J. Leg. Med..

[9] Rokhshad R., Nasiri F., Saberi N., Shoorgashti R., Ehsani S.S., Nasiri Z., Azadi A., Schwendicke F. (2025). Deep learning for age estimation from panoramic radiographs: A systematic review and meta-analysis. J. Dent..

[10] Bizjak Ž., Robič T. (2024). DentAge: Deep learning for automated age prediction using panoramic dental X-ray images. J. Forensic Sci..

[11] Gandikota C.S., Rayapudi N., Challa P.L., Juvvadi S.R., Yudhister P.V., Rao G.H. (2012). A comparative study of linear measurements on facial skeleton with frontal and lateral cephalogram. Contemp. Clin. Dent..

[12] Takeda S., Mine Y., Yoshimi Y., Ito S., Tanimoto K., Murayama T. (2021). Landmark annotation and mandibular lateral deviation analysis of posteroanterior cephalograms using a convolutional neural network. J. Dent. Sci..

[13] Ito S., Mine Y., Urabe S., Yoshimi Y., Okazaki S., Sano M., Koizumi Y., Peng T.Y., Kakimoto N., Murayama T. (2024). Prediction of a Cephalometric Parameter and Skeletal Patterns from Lateral Profile Photographs: A Retrospective Comparative Analysis of Regression Convolutional Neural Networks. J. Clin. Med..

[14] Ramírez-Sotelo L.R., Almeida S., Ambrosano G.M., Bóscolo F. (2012). Validity and reproducibility of cephalometric measurements performed in full and hemifacial reconstructions derived from cone beam computed tomography. Angle Orthod..

[15] Baldini B., Cavagnetto D., Baselli G., Sforza C., Tartaglia G.M. (2022). Cephalometric measurements performed on CBCT and reconstructed lateral cephalograms: A cross-sectional study providing a quantitative approach of differences and bias. BMC Oral. Health.

[16] Suomalainen A., Pakbaznejad Esmaeili E., Robinson S. (2015). Dentomaxillofacial imaging with panoramic views and cone beam CT. Insights Imaging.

[17] Devlin H., Yuan J. (2013). Object position and image magnification in dental panoramic radiography: A theoretical analysis. Dentomaxillofac. Radiol..

[18] Zhang Z., Liu N., Guo Z., Jiao L., Fenster A., Jin W., Zhang Y., Chen J., Yan C., Gou S. (2022). Ageing and degeneration analysis using ageing-related dynamic attention on lateral cephalometric radiographs. NPJ Digit. Med..

[19] Pecora N.G., Baccetti T., McNamara J.A. (2008). The aging craniofacial complex: A longitudinal cephalometric study from late adolescence to late adulthood. Am. J. Orthod. Dentofac. Orthop..

[20] Van’t Spijker A., Rodriguez J.M., Kreulen C.M., Bronkhorst E.M., Bartlett D.W., Creugers N.H. (2009). Prevalence of tooth wear in adults. Int. J. Prosthodont..

[21] Kushchayev S.V., Glushko T., Jarraya M., Schuleri K.H., Preul M.C., Brooks M.L., Teytelboym O.M. (2018). ABCs of the degenerative spine. Insights Imaging.

[22] Hase H., Mine Y., Okazaki S., Yoshimi Y., Ito S., Peng T.Y., Sano M., Koizumi Y., Kakimoto N., Tanimoto K. (2024). Sex estimation from maxillofacial radiographs using a deep learning approach. Dent. Mater. J..

[23] Huang G., Liu Z., van der Maaten L., Weinberger K.Q. (2016). Densely connected convolutional networks. arXiv.

[24] Selvaraju R.R., Cogswell M., Das A., Vedantam R., Parikh D., Batra D. (2020). Grad-CAM: Visual explanations from deep networks via gradient-based localization. Int. Comput. Vis..

[25] Saporta A., Gui X., Agrawal A., Pareek A., Truong S.Q.H., Nguyen C.D.T., Ngo V.D., Seekins J., Blankenberg F.G., Ng A.Y. (2022). Benchmarking saliency methods for chest X-ray interpretation. Nat. Mach. Intell..

[26] Patcas R., Wiedemeier D.B., Markic G., Beit P., Keller H. (2017). Evidence of secular trend in mandibular pubertal growth. Eur. J. Orthod..

[27] Franchi L., Nieri M., Lomonaco I., McNamara J.A., Giuntini V. (2021). Predicting the mandibular growth spurt. Angle Orthod..

[28] Oliveira W., Albuquerque Santos M., Burgardt C.A.P., Anjos Pontual M.L., Zanchettin C. (2024). Estimation of human age using machine learning on panoramic radiographs for Brazilian patients. Sci. Rep..

[29] Mu C.C., Li G. (2022). Age Estimation using Panoramic Radiographs by Transfer Learning. Chin. J. Dent. Res..

[30] Fan F., Ke W., Dai X., Shi L., Liu Y., Lin Y., Cheng Z., Zhang Y., Chen H., Deng Z. (2023). Semi-supervised automatic dental age and sex estimation using a hybrid transformer model. Int. J. Leg. Med..

